# RESEARCH BEST PRACTICES TO SUPPORT RESILIENCE IN EYE STROKE VISION REHABILITATION

**DOI:** 10.1093/geroni/igad104.3573

**Published:** 2023-12-21

**Authors:** Kimberly Hreha, Nathan Boucher, Heather Whitson

**Affiliations:** Duke University, Durham, North Carolina, United States; Veterans Health Administration, Durham, North Carolina, United States; Duke University, Durham, North Carolina, United States

## Abstract

While effective rehabilitation exists to promote independence and community participation for age-related vision impairment, no rehabilitation program has been developed specifically for eye stroke impairment. We are adapting a macular degeneration self-management program using input from key partners to inform future intervention. The pilot phase objective was to inform future study design for eye stroke survivors. We conducted semi-structured interviews, and applied the 5T Framework (Target population identification, Team composition, Time considerations, Tips to accommodate older adults, and Tools for inclusive enrollment of older adults) to develop the interview guide. Twenty-five people recruited from clinical settings were consented/interviewed over six months. This included 10 stroke survivors (M age= 66 years; 70% male--90% visual field defect), 4 care partners (M age= 57 years; 100% female--1 mother, 1 sister and 2 spouses), and 11 providers (M age= 37 years; 54.5% female--3 occupational therapists, 4 ophthalmologists, 2 optical technicians, 1 optometrist, 1 physical therapist). The most meaningful outcomes identified were driving and returning to full independence. The most frequently identified “facilitator of recovery” after eye stroke was family support. The most frequently identified barriers to participation in intervention research were lack of accessibility of the research building and transportation. Optimal lighting and scanning strategies were identified as key elements of any future intervention as well as approaches to support enrollment and retention in study activities. Interview data are now being used to inform phase two: adaptation of the self-management program and design of the study that will evaluate its effectiveness.

